# Supplementary material for: Risk Factors for Early Hospital Readmission in Geriatric Patients: A Systematic Review
Source: Int J Environ Res Public Health. 2023 Jan 17;20(3):1674. doi: 10.3390/ijerph20031674 (PMC9914102; doi:10.3390/ijerph20031674)
Supplement: Supplementary file 1 [file ijerph-20-01674-s001.zip › ijerph-2125706-supplementary.pdf]

Table S1. Studies included, design, variables considered and results.

Study	Study type	Data source	Clinical setting	Study population and sample size	Analytic model and outcome	Variables	Results
**Van Seben et al. 2020**	Prospective cohort study	Questionnaires and comprehensive geriatric assessment at admissions, discharge, 1 month, 2 months and 3 months post discharge.	Multicentric study in University Medical Centre and five regional teaching hospitals in the Netherlands.	Patients aged ≥ 70 years admitted at Internal Medicine, Cardiology or Geriatric wards.<br><br>Sample size : 401	Analytic model : logistic regression<br><br>Outcome: functional decline, unplanned readmission and mortality at 1, 2 and 3 months post discharge.	<u>Socio-demographics</u> : age, gender, stay of life, marital status, patients' educational level, country of born, discharge destination<br><u>Health characteristics</u> : CCI, hearing impairment, vision impairment, MMSE, GDS-15, GDS-3 for apathy, NRS, SNAQ, continence item on the Katz-ADL, Mobility impairment, fall in the past 6 months prior to hospitalization, during the hospitalization and in the first, second and third month post discharge.<br><u>Health care utilization</u> : Prior admission within 6 months of indexed admission, length of hospital stay, received ambulatory physiotherapy within 3 months post discharge.<br><u>Clinical conditions</u> : patient's diagnoses, medication	-Malnutrition<br>OR 1.80 (1.21-2.68)<br>-Developing cognitive impairment at 1 month post discharge<br>OR 6.40 (1.52–26.84)<br>-Developing fatigue at 1 month post discharge<br>OR 4.71 (1.03–21.60)<br>-Developing fall risk at 1 month post discharge<br>OR 4.30 (1.21–16.57)<br><br>Rate of readmission within 3 month post discharge: 33.9 %<br><br>Rate of readmission during the second or third month post discharge: 19 %
**Calsolaro et al. 2019**	Retrospective cohort study	Questionnaires and comprehensive geriatric assessment at admissions,	Monocentric study in Geriatrics Unit of the University	Patients admitted to Geriatrics Unit<br><br>Sample size: 1434	Analytic model : multivariate logistic regression<br><br>Outcome: 30 days potentially	<u>Socio-demographics</u> : sex, age<br><u>Health characteristics</u> : CIRS, ADL, IADL, SPMSQ, MNA, CAM	-Disability (ADL score 0-1)<br>OR 2.06 (1.34-3.16)<br>-Severity of comorbidity (CIRS-S > 2.38)<br>OR 1.73 (1.02-2.93)<br>-Diagnosis of sepsis<br>OR 2.56 (1.31-5.00)

		medical records, hospital discharge register	Hospital of Pisa (Italy)		preventable readmissions	Health care utilization : length of hospital stay<br>Clinical conditions : patient's diagnosis	Rate of readmission : 11,9 % potentially preventable readmission
**Lin et al. 2019**	Quasi-experimental study	Clinical screening (CAM, CGS et Mini-Cog) at admission. Medical charts review and telephone calls for readmissions	Monocentric study in geriatric ward at National Taiwan University Hospital.	Patients admitted to Geriatrics Unit<br><br>Sample size : 377 CGS group et 380 control.	Analytic model : Multivariate analysis performed using the Cox proportional regression model.<br><br>Outcome: unexpected patient readmission within 30 days after discharge.	Socio-demographics : sex, age, marital status, education<br>Health characteristics : CCI, Barthel index score at admission, early comprehensive geriatric screen<br>Health care utilization : length of hospital stay, N of admission<br>Clinical conditions : comorbidities	-CCI<br>HR 1.64 (1.09-2.46)<br>-Number of admissions ≥ 4 within 1 year before admission date<br>HR 2.92 (1.49-574)<br><br>Rate of readmission:<br>11.4% in CGS group et 16.9 in control group
**Lehn et al. 2019**	Observational prospective study	Clinical screening and Danish National Patient Register	Monocentric study in department of medicine at Holbaek University Hospital (Denmark). February to September 2012	Patients aged ≥ 65 years admitted at department of medicine (including the medical specialities of internal medicine)<br><br>Sample size : 770	Analytic model : descriptive univariate logistic regression analyses and logistic regression model selection with significance level at 10%<br><br>Outcome: 30 days acute all-cause readmissions	Socio-demographics :<br>age, gender, patients' educational level, patients' income, patients living alone or not, CCI of the spouse, children's residence<br>Health characteristics : ADL<br>Health care utilization :<br>contact with general practitioners and the after-hours emergency service doctor 1 month before indexed admission, prior admission within 3 months of indexed admission, distance between patient home and general practitioner clinic, length of hospital stay, previous admissions	-Having primary school as highest level of education<br>OR 1.79 (0.94 to 3.38)<br>-Male<br>OR 1.71 (1.15 to 2.54)<br>-Contact with emergency service doctor within 1 month<br>before index admission<br>OR 1.43 (0.99 to 2.09)<br>-Primary diagnosis related to atypical symptoms<br>OR 0.64 (0.39 to 1.03)<br>-CCI score 4+<br>OR 1.88 (1.63 to 3.04)<br>-LOS 6+<br>OR 1.47 (1.01 to 2.15)<br>-Clinical assessment of cognitive problems<br>OR 2.19 (1.10 to 4.36)<br>-Medication for glaucoma<br>OR 2.20 (1.02 to 4.78)<br>-Medication for acid disorders

						<u>Clinical conditions</u> : patient's diagnoses, medication	OR 1.89 (1.29 to 2.77)<br>-Medication for thyroid disease<br>OR 1.83 (0.94 to 3.57)<br><br>Rate of readmission: 20 %
**Chan et al. 2019**	Prospective observational cohort study	Administrative records of different hospitals. Follow up with call at patients by phone	Monocentric study in San Francisco General Hospital (USA)<br>July 2010-August 2012	Patients aged ≥ 55 years admitted at department of medicine (medicine, cardiology or neurology services). Excluded patients with planned hospitalization, likely to be discharged to an institutional setting, unable to consent, diagnosed with metastatic cancer and unable to participate in telephone follow-up.<br>Sample size: 674	Analytic model : multivariable logistic regression.<br><br>Outcome: 30 days unplanned readmissions	<u>Socio-demographics</u> : age, gender, ethnicity, patients' educational level, total household income, English proficiency, patients living alone or not, health literacy<br><u>Health characteristics</u> : ADL, CCI, substance use, depression, cognitive impairment.<br><u>Health care utilization</u> : prior admission within 6 months of indexed admission	-Latino/Hispanic ethnicity<br>OR 0.31 (0.12 to 0.77)<br>-CCI ≥ 5<br>OR 4.19 (1.59 to 11.07)<br><br>Rate of readmission: 14.3%
**Maddox et al. 2019**	Retrospective cohort study	Medicare 100 percent Research Identifiable Files (RIF)	Multicentric study including hospitals subject to the Medicare's Hospital Readmissions Reduction Program (HRRP)	Fee-for-service Medicare beneficiaries with acute myocardial infarction (AMI), congestive heart failure (CHF) or pneumonia from December 2012 to November 2015.<br><br>Sample size: 7907	Multivariate analysis using a generalised linear models<br><br>Outcome: 30 days unplanned readmissions	<u>Socio-demographics</u> <u>:</u> sex, age, race, housing stability, patient's neighbourhood disadvantage by ADI, hospital population residing in highly disadvantaged neighbourhoods by ADI<br><u>Health characteristics</u> : Mean number of comorbidities, disability<br><u>Health care utilization</u> : Medicaid	<u>AMI cohort</u><br>-Medicaid<br>OR 1.09 (1.07-1.12)<br>-Disability<br>OR 1.10 (1.07-1.12)<br>-Hospital population residing in highly disadvantaged neighbourhoods by ADI<br>OR 1.04 (1.01-1.07)<br><br><u>Pneumonia cohort</u><br>-Medicaid<br>OR 1.14 (1.13-1.16)<br>-2 ou 3 ZIPs

							OR 1.04 (1.03-1.06)<br>-4 or more ZIPs<br>OR 1.28 (1.19-1.38)<br>-Patient's neighbourhood disadvantage by ADI<br>OR 1.04 (1.01-1.07)<br>-Hospital population residing in highly disadvantaged neighbourhoods by ADI<br>OR 1.02 (1.01-1.04)<br><br>CHF cohort<br>-Medicaid<br>OR 1.16 (1.15-1.17)<br>-Disability<br>OR 1.10 (1.09-1.11)<br>-2 ou 3 ZIPs<br>OR 1.07 (1.05-1.08)<br>-4 or more ZIPs<br>OR 1.29 (1.20-1.38)<br>- Hospital population residing in highly disadvantaged neighbourhoods by ADI<br>OR 1.05 (1.03-1.07)<br><br>Rate of readmission:<br>- AMI cohort : 14.7%<br>- Pneumonia cohort : 15.2%<br>- CHF cohort :19.6%
**Cotter et al. 2012**	Retrospective cohort study	Hospital data system	Monocentric study in department of Medicine for Elderly at Cambridge University Hospitals. January-December 2010	All patients admitted to department of Medicine for Elderly and discharged to the local Primary Care Trust.<br><br>Sample size: 507	Multivariate analysis using a logistic regression model<br><br>Outcome: 30 days readmissions, death, death or readmission.	Socio-demographics : sex, age<br>Health characteristics : CCI<br>Health care utilization : length of hospital stay, number of previous emergency department attendances within 6 month	-Previous ED visits<br>[Exp(B) = 1.315; P = 0.001]<br><br>Rate of readmission : 17.8%

**Uhlmann et al. 2017**	Retrospective cohort study	Hospital data system	Monocentric study in CHUV (Switzerland) January 2009-Decembre 2011	All patients admitted to division of general internal medicine at CHUV<br><br>Sample size: 6729	Bivariate analysis and multivariate regression<br><br>Outcome: 30 days potentially avoidable readmission	Socio-demographics : sex, age, where the patient came from on admission, where the patient went to at discharge<br>Health characteristics : CCI<br>Health care utilization : length of hospital stay, number of admissions to the CHUV at 6 and 12 months beforehand<br>Clinical conditions : patient's diagnosis, blood sodium, blood haemoglobin, blood creatinine, blood urea, blood albumin, serum alanine aminotransferase, number of medications prescribed during the stay and number of medications prescribed at discharge, number of different prescribers during the stay	-At least one hospitalisation in the 12 months preceding the index admission<br>OR 1.96 (1.67 to 2.31)<br><br>-Cancer diagnosis with metastasis<br>OR 3.14 (2.46 to 4.00)<br>-Cancer diagnosis without metastasis and without antidepressant<br>OR 1.81 (1.39 to 2.36)<br>-Blood sodium <135 mmol/l<br>OR 2.15 (1.65 to 2.80)<br>-CCI score >1<br>OR 1.46 (1.21 to 1.76)<br>-LOS >11 days<br>OR 1.36 (1.15 to 1.61)<br>-Prescription of at least 15 different medications during the stay<br>OR 1.27 (1.06 to 2.52)<br><br>Rate of readmission : 11.5% potentially preventable readmission
**Anderson et al. 2016**	Prospective observational cohort study	Clinical screening, questionnaires and Hospital data system	Monocentric study in Barnes Jewish Hospital (USA) January 2012-Decembre August 2013	Patients aged ≥ 65 years discharged to home from the medicine service in Barnes Jewish Hospital<br><br>Sample size: 381	Analytic model : logistic regression model<br><br>Outcome: 30 days all cause readmissions	Socio-demographics : sex, age, race, living alone<br>Health characteristics : Short Blessed Test score, Trail-Making Test part B, Patient Health Questionnaire-2 depression score, number of medications prescribed at discharge, medication nonadherence, independent in medication management<br>Health care utilization : LOS<br>Clinical conditions : patient's diagnosis	-Short Blessed Test >12 and independence in own medication management<br>RR 3.7 (P=0.006)<br>-Trail-Making Test Part B non-completed and independence in own medication management<br>RR 2.8 (P=0.007)<br><br>Rate of readmission : 21.8%

**Bogaisky et al. 2015**	Retrospective cohort study	Hospital register	Multicentric study (three-hospitals of Montefiore Medical Center in New-York) USA	Patients aged ≥ 65 years admitted to the geriatric inpatient service at Montefiore's Moses Division with readmission to any of Montefiore's three hospitals. All patients are nursing home residents (60%) or lived in the community (40%)<br><br>Sample size: 1038	Analytic model : logistic regression<br><br>Outcome: 30 days any readmissions	<u>Socio-demographics</u> : sex, age<br><u>Health care utilization</u> : residence location before admission and discharge location, discharging physician<br><br><u>Clinical conditions</u> : primary discharge diagnosis, medical comorbidities	Results community dwellers :<br>- COPD OR 2.0 (1.2-3.3)<br>- Renal failure OR 2.7 (1.7-4.3)<br>- Pressure ulcer OR 3.5 (1.7-7.1)<br><br>Results nursing home residents :<br>- CHF OR 1.5 (1.1-2.1)<br>- Dementia OR 1.4 (1.1-2.0)<br>- Pressure ulcer OR 1.6 (1.2-2.2)<br>- Renal failure OR 1.4 (1.0-1.9)<br>- Hypertension OR 0.7 (0.4-0.96)<br>- Discharging physician is a hospitalist geriatrician OR 0.7 (0.5-0.9)<br><br>Rate of readmission: 34.4% for nursing home residents and 22.6% for community dwellers ($p<0.001$)
**Scott et al. 2014**	Retrospective case-control study	Medical records	Monocentric study in general medicine service at Brisbane (Australia)	Patients aged ≥ 65 years admitted to general medicine service<br><br>Sample size: 311<br>(Case = 113, Control = 198)	Variables were compared between readmissions and controls using chi-square tests, t-test and Mann–Whitney U-tests for proportions, normally and non-normally distributed data, respectively, with P-values <0.05 denoting statistical significance.<br><br>Outcome: 30 days unexpected readmissions	<u>Socio-demographics</u> : sex, age, non-English speaking, marital status, residing in nursing home<br><u>Health characteristics</u> : comorbidity score, medications at index discharge, level of functional impairment on presentation index admission<br><u>Health care utilization</u> : length of hospital stay, admission within 6 months prior, Quality of care factor<br><u>Clinical conditions</u> : patients' diagnosis at index admission	-Higher comorbidity burden<br>P=0.003<br>-Non English speaking<br>P=0.046<br>-One or more hospital admissions over the 6 months preceding index admission<br>P<0.001<br>-One or more quality of care<br>P<0.001<br><br>Rate of readmission: not calculated.

**Fisher et al. 2012**	Prospectiv e cohort study	Medical records, nurse assessment sheets, telephone call during the first week post-discharge	Monocentric study in acute Care for Elders unit at University of Texas Medical Branch	Patients aged ≥ 65 admitted to acute care for elders unit (for medical illness). Patients able to walk safely, discharged directly to home<br><br>Sample size: Participants n : 111	Analytic model: logistic regression<br><br>Outcome: any unplanned hospitalization back to same ospital within 30 days	<u>Socio-demographics</u> : age, gender, ethnicity, marital status<br><u>Health characteristics</u> : BMI, comorbidity, cane or walker prior to admission, ADL limitations, mobility level after discharge: daily steps, mobility change first to last day, mobility rate of change<br><u>Clinical conditions</u> : reasons for admission (diagnosis), in hospital severity of illness<br><u>Health care utilization</u> : admitted and discharged for 24-h observation, LOS	None significant in multivariable analysis<br><br>Rate of readmission: 11.7%
**Franchi et al. 2012**	Prospectiv e cohort study	Questionnaires at admissions, medical records, follow up with call at patients by phone	Multicentric study. Data from 66 hospitals participating in the « REPOSI » Italy	Patients aged ≥ 65 years admitted to internal and geriatric medicine wards and with readmission<br><br>Sample size : 766	Univariate logistic regression. The variables clinically and statistically significant are included in the multivariate model<br><br>Outcome: 3 months readmissions	<u>Socio-demographics</u> : sex, age, educational background, marital status, body mass index, living arrangement<br><u>Health characteristics</u> : cognitive status (Short Blessed Test), GDS, Barthel ADL index, smokers, drinker<br><u>Health care utilization</u> : history of previous admission in the previous 6 months, length of hospital stay<br><u>Clinical conditions</u> : CIRS, number of diagnoses and drugs at discharge, adverse clinical events during hospitalization	-Adverse clinical events during hospitalization OR 1.74 (1.19-2.56)<br>-Admission in the previous 6 months OR 1.92 (1.31-2.82)<br>-Cardiovascular diseases (level of impairment ≥3) OR 1.48 (1.00-2.17)<br>-Liver diseases (level of impairment ≥3) OR 2.32 (1.42-3.77)<br><br>Rate of readmission: 19% within 3 months

**Ben-Chetrit et al. 2012**	Retrospective cohort study	Discharge letters	Multicentric study in three Hospitals in Jerusalem (Israel). January 2008-December 2009.	Patients aged ≥ 65 years admitted to internal medecine wards and with unlanned readmission.<br><br>Sample size: 496	Multivariate logistic regression analysis<br><br>Outcome: 3 months readmissions	Socio-demographics : sex, age, residence (home, nursing institution)<br>Health characteristics : cognitive status<br>Health care utilization : number of admissions during 2008-2009, length of hospital stay<br>Clinical conditions : major diagnoses on the discharge letter, laboratory data	-Age 80 years or older*<br>-Impaired cognition*<br>-Nursing home residence*<br>-CHF*<br>-Creatinine level above 1.5 mg/dl*<br><br>Rate of readmission: 58% within 3 months
**Zapatero et al. 2012**	Retrospective cohort study	National discharge records of Internal medicine (Basic Minimum Data Sets)	Multicentric study with data of all departments of Internal Medicine. January 2006-December 2007, Spain.	All patients admitted to the medical ward and with unplanned readmission within 30 days<br><br>Sample size : 999,089	Analytic model : multivariable logistic regression<br><br>Outcome: 30 days unplanned readmissions	Socio-demographics : sex, age<br>Health characteristics : comorbidities<br>Health care utilization : LOS, day of discharge<br>Clinical conditions : CCI, Major Diagnostic Category	-LOS*<br>-Male sex*<br>-Acute congestive heart failure*<br>-Exacerbation of COPD*<br>-Severe liver disease*<br>-Anaemia*<br>-Malnutrition*<br>-Diabetes*<br>-Dementia*<br>-Chronic renal insufficiency*<br>-Cancer*<br>-Atrial fibrillation*<br><br>Rate of readmission: 12.4%
**Fitriana et al. 2021**	Prospective cohort study	Questionnaires at admissions, medical records, follow up with call at patients by phone	Monocentric study in Cipto Mangunkusumo Hospital (Indonesia)	Patients aged ≥ 60 years admitted to the acute care ward and with unplanned readmission<br><br>Sample size : 266	Analytic model : logistic regression<br><br>Outcome: 30 days unplanned readmissions	Socio-demographics : sex, age, educational background, marital status, living status, caregiver, income<br>Health characteristics : FRAIL scale, Barthel ADL index, MNA-SF, AMT, GDS-15<br>Health care utilization : history of previous	-MNA-SF/point<br>OR 2.01 (1.07-4.11)<br>-GDS-15/point<br>OR 1.88 (1.02-3.44)<br><br>Rate of readmission : 37.6%

						admission in the previous 6 months, length of hospital stay<br>Clinical conditions : severity of diseases (ICD-10), presence of malignancy, CCI, the amount of prescribed medication	
**Robinson et al. 2012**	Retrospective cohort study	Data from Ministry of Health's National Minimum Data Set	Multicentric study. Data from all hospitals in New Zealand between 1.2.2009 and 31.06.2010	Patients aged ≥ 65 years with acute medical Admissions and a subsequent routine or self discharge<br><br>Sample size: participants n = 66,982 persons representing n = 95,318 admissions	Analytic model: binominal regression<br><br>Outcome: acute medical readmissions within 30 days and 90 days of discharge	Socio-demographics : age, gender, ethnicity, socioeconomics deprivation (NZDep06-Index), rurality<br>Clinical conditions : diagnoses, admission complexity (not included as covariates in the risk analysis)	Results for readmissions within 30 days :<br><br>-Age (years over 65):<br>RR 1.026 (1.016–1.036); P < 0.001;<br>-Male gender:<br>RR 1.161 (1.127–1.197); P < 0.001;<br>-Ethnicity<br>(compared with European): Maori<br>RR 1.179 (1.115–1.246); P < 0.001;<br>-Pacific<br>RR 1.283 (1.203–1.370); P < 0.001;<br>-Living conditions: living in deprived areas: NZDep06-Index 4–7:<br>RR 1.043 (1.000–1.087); P = 0.049; NZDep06-Index 8–10:<br>RR 1.152 (1.104–1.203); P < 0.001;<br>-Rurality:<br>RR: 0.891 (0.839–0.946); P < 0.001<br><br>Rate of readmission:<br>10.8% by 30 days after discharge, 18.3% by 90 days after discharge.
**Shu et al. 2012**	Retrospective cohort study	Hospital data system	Monocentric study in general medical ward of the National	Patients aged ≥ 16 years and admitted to the general medical ward from the ED. Patients who died in	Analytic model : multivariate cox proportional hazard regression<br><br>Outcome : 30 days all readmissions	Socio-demographics : sex, age, educational status, marital status<br>Health characteristics : CCI, Barthel score on admission<br>Health care utilization : LOS	-CCI score 2–4<br>HR 1.42 (1.07–1.89)<br>-CCI score >4<br>HR 1.93 (1.37–2.73)<br>-Underlying active malignancy<br>HR 1.66 (1.27–2.16)<br>-LOS, 8-14 days

			Taiwan University Hospital November 2009-April 2010	the hospital or went home to palliative care after discharge were excluded.<br>Average age : 67 years.<br><br>Sample size:<br>2932		Clinical conditions : underlying chronic illnesses, presence of anaemia	HR 1.51 (1.17–1.95)<br>-LOS 15–28 days<br>HR 1.64 (1.22–2.19)<br>-LOS >28 days<br>HR 1.97 (1.43–2.71)<br>-Anaemia<br>HR 1.26 (1.02–1.55)<br><br>Rate of readmission: 16.7%
**Finlayson et al. 2018**	Prospective Case-Control trial	Hospital medical records, patients interviews	Multicentric study in two metropolitan hospitals in Australia.<br>Data collection from 2008 to 2011	Patients aged ≥ 65 years with medical admission and at least one risk factor for readmission.<br>Excluded patients requiring home oxygen, unable to walk independently for 3 m, living in a nursing home or with cognitive deficit.<br><br>Sample size: 222	Analytic model : Cox proportional hazards regression mode<br><br>Outcome: 28 days, 12 weeks and 24 weeks unplanned readmissions	Socio-demographics : sex, age, educational background, employment status, income, living arrangements, hospital insurance status.<br>Health characteristics : IADL index, Walking Impairment Questionnaire, GDS, SF-12, MOS Social Support Survey, Number of risk factors of readmission, history of depression<br>Health care utilization : history of previous admission in the previous 30 days and 6 months, length of hospital stay<br>Clinical conditions : Admission diagnosis, comorbidities	•28 days readmission:<br>-Co-existing renal disease<br>HR 2.66 (1.18-5.97)<br>-Higher GDS score<br>HR 1.91 (1.03-1.37)<br>-Higher score at Chronic Disease Self Efficacy Scale<br>HR 1.59 (1.20-2.11)<br><br>Rate of readmission:14.2%<br><br>•12 weeks readmission:<br>Rate of readmission :28%<br>No statistically significant risk factors
**Higi et al. 2021**	Retrospective cohort study	Hospital data system	Monocentric study in general medical ward of the teaching hospital in Baden (Switzerland).	Patients aged ≥ 65 years with medical admission.<br><br>Sample size: 5985	Analytic model : multivariable logistic regression<br><br>Outcome: 30 days potentially avoidable readmissions	Socio-demographics : sex, age<br>Health care utilization : history of previous admission in the previous 6 months, length of hospital stay<br>Clinical conditions :	-Admission in previous 6 months<br>OR 1.39 (1.08-1.77)<br>-Anaemia<br>OR 1.45 (1.12-1.85)<br>-Heart failure<br>OR 1.41 (1.09-1.81)<br>-Opioids<br>OR 1.4 (1.1-1.78)

			December 2016-November 2018			number of drugs dispensed, opioids use, hyperkalaemia, comorbidities	Rate of readmission : 5.7% potentially preventable readmission
**Vallini et al. 2021**	Retrospective case-control study	Electronic clinical chart and hospital discharge register	Monocentric study in Internal medicine unit of the hospital of Pontedera (Italy). January 2018-December 2018	All patients admitted in the internal medicine unit of the hospital of Pontedera.<br><br>Sample size: 3012	Analytic model : independent sample t-test for the continuous variables, $\chi 2$ test for categorical variables. Significance was inferred for a P value <.05<br><br>Outcome: 30 days unplanned readmissions	Socio-demographics : sex, age, patient setting before index hospitalization<br>Health care utilization : length of hospital stay<br>Clinical conditions : comorbidities	-Age p 0.000018<br>-Cerebrovascular disease p<0.00001<br>-Cognitive impairment p<0.00001<br>-CHF p<0.00001<br>-Chronic kidney disease p<0.00001<br>-COPD p 0.001594<br>-Skin ulcers p<0.00001<br>-Extended care facilities before index hospitalization P 0.001267<br><br>Rate of readmission : 14.1%
**Mueller et al 2021**	Retrospective cohort study	National register: Federal statistical office of Switzerland	Multicentric study with data concerning all adult medical inpatients in Switzerland from January 2012 to December 2017	All adult medical inpatients discharged in Switzerland.<br><br>Sample size: 1,463,781	Analytic model : multivariable logistic regression model<br><br>Outcome: 30 days and 1 year all cause readmissions	Clinical conditions : comorbidities	-Multimorbidity (co-occurrence of two or more chronic health conditions)<br>OR 1.92 (1.89-1.94)<br><br>Rate of readmission : 11.5%
**Oud et al 2021**	Retrospective cohort study	Hospital files and discharge letter	Monocentric study in geriatric ward of a teaching hospital in Netherlands in 2015	Patients aged ≥ 70 years with medical admission.<br><br>Sample size: 5985	Analytic model : univariate logistic regression model<br><br>Outcome: 3-month all cause readmissions	Health characteristics :VMS, delirium risk, fall risk, malnutrition, physical impairment	-Malnutrition<br>OR 1.74 (1.08-2.91)<br><br>Rate of readmission : 16%
**Wang-Hansen et al**	Prospective cohort study	Referral letter, electronic patient records	Monocentric study in medical	Patients aged ≥ 75 years, receiving	Analytic model : adjusted proportional	Socio-demographics : sex, age<br>Health characteristics :	-Female gender<br>OR 2.17 (1.15-4.00)<br>-Lower age

**2022**		and patient administrative system	department at a Norwegian regional hospital.<br>1 April – 31 October 2012	home care services, with medical admission. Patients lived in a long-term care facility were excluded.<br><br>Sample size : 227	hazards Cox regression model<br><br>Outcome: 30 days all cause readmissions	CIRS-G, Barthel ADL, MMSE, eGFR, number of daily drugs<br>Health care utilization : hospitalisation ward	OR 0.95 (0.91-0.99)<br>-Higher MMSE score<br>OR 1.03 (1.00-1.06)<br><br>Rate of readmission : 26%
**Goldin et al 2018**	Retrospective cohort study	Electronic health records	Monocentric study in medicine service of tertiary centre in New York (USA)<br>May 2014 - June 2015	Patients aged ≥ 75 years with medical admission.<br><br>Sample size:<br>3751	Analytic model : Chi-squared test to evaluate the association between the categorical variables.<br><br>Primary outcome: non-compliance, defined as the absence of an order for VTE prophylaxis for the duration of hospitalization or an interruption of prophylaxis exceeding 24 h.<br><br>Secondary outcome: 30 days all readmissions	Clinical conditions : VTE prophylaxis non-compliance	-VTE prophylaxis non-compliance<br>P<0.001<br><br>Rate of readmission : 14.16% total, 13.45% compliant, 19.85% non-compliant
**Kim et al 2016**	Retrospective cohort study	Cleveland Clinic Health System administrative data and electronic medical records	Multicentric study in Cleveland Clinic Health System including the main campus and eight	All admissions to Cleveland Clinic Main Campus medicine services discharged to Skilled nursing facilities (SNF).	Analytic model : univariate analyse<br><br>Outcome: 30 days all cause readmissions	Socio-demographics : sex, age, race<br>Health characteristics : Medicare severity diagnosis-related group<br>Health care utilization : history of previous admission in the previous 1	-Lower age<br>P<0.0001<br>-Haemodialysis<br>P<0.0001<br>-Discharged from a subspecialty rather than general medicine services<br>P<0.0001

			regional community hospitals (USA).<br>January 2011 – December 2012	Sample size:<br>4208		year , length of hospital stay, department of stay<br>Clinical conditions : Hb on discharge, Na on discharge, comorbidities, haemodialysis	-Higher Medicare severity diagnosis-related group<br>P<0.0001<br>-Longer LOS<br>P<0.0001<br>-More comorbidities (myocardial infarction, CHF, peripheral vascular disease, COPD, diabetes, chronic kidney disease and liver disease)<br>P<0.0001<br><br>Rate of readmission : 30.9%
**Silber et al 2019**	Case-control study	Centres for Medicare & Medicaid Services (CMS) Virtual Research Data Centre (VRDC) to administrative claims data for older Medicare beneficiaries	Multicentric study in 2872 hospitals in USA.<br>July 2012-November 2014	Medicare patients at 339 major teaching hospitals matched patient controls from 2439 non-teaching hospitals. Hospitalization for AMI, HF, and PNA.<br><br>Sample size: 1.252.959	Analytic model : univariate analyse with McNemar's test<br><br>Outcome: 30 days all cause readmissions	Health care utilization : hospitalization in major teaching hospital or in non-teaching hospital.	-Hospitalization in non-teaching hospital<br>P<0.0001<br><br>Rate of readmission : 21.6% in major teaching hospital, 22.5% in non-teaching hospital
**Fisher et al 2016**	Prospective cohort study	Electronic health record of the hospital	Monocentric study in university teaching hospital in USA	Patients aged 65 years and above admitted for the following medical diagnoses: cardiovascular, pulmonary, infection, gastrointestinal or endocrine.<br><br>Sample size: 164	Analytic model: multivariate logistic regressions models<br><br>Outcome : 30 days unplanned readmissions	Health characteristics : ADL, Walking activity (main daily steps)	-Main daily steps<br>OR 0.89 (0.82-0.98)<br><br>Rate of readmission : 15.8%
**Froom et al 2020**	Cohort study	Electronic health record of the hospital	Monocentric study in internal	All acutely hospitalized patients who were	Analytic model: multivariate logistic	Socio-demographics: age<br>Health care utilization : LOS, time from last	-Age*<br>OR 1.30 (1.22-1.39)<br>-Previous number hospitalizations*

			medicine and cardiology departments at Laniodo hospital in Israel.<br><br>2015-2016 and January-October 2019	discharged from internal medicine and cardiology departments.<br><br>Sample size: 27289 (18475 in 2015-2016 and 8814 in 2019)	regressions models<br><br>Outcome: 30 days readmissions	hospitalization, number previous hospitalizations<br>Clinical conditions :<br>Discharge diagnosis of: congestive heart failure, chronic obstructive lung disease, non-specific chest pain, syncope, haemoglobin, albumin, platelets, blood urea nitrogen, serum sodium	OR 1.37 (1.30-1.45)<br>-Last hospitalization <180 days*<br>OR 1.24 (1.18-1.30)<br>-Discharge diagnosis of*:<br>--CHF<br>OR 1.27 (1.10-1.48)<br>--COPD<br>OR 1.46 (1.20-1.78)<br>--haemoglobin ≤ 12 g/l<br>OR 1.08 (1.01-1.16)<br>--albumin<br>OR 1.25 (1.16-1.33)<br>--serum sodium ≤ 135 meq<br>OR 1.20 (1.08-1.34)<br>--blood urea nitrogen ≥ 20 mg/dl<br>OR 1.19 (1.12-1.26)<br>--platelet count > 300 10^9/L<br>OR 1.15 (1.07-1.24)<br>Rate of readmission*: 13.7%<br><br>* Data of the cohort 2015-2016

Odds ratio (OR), Relative risk (RR), Hazard ratio (HR), Charlson Comorbidity Index (CCI), Mini-mental state examination (MMSE), Geriatric Depression Scale-15 (GDS-15), Geriatric Depression Scale-3 (GDS-3), Numerating Rating Scale (NRS), Short Nutritional Assessment Questionnaire (SNAQ), Cumulative Illness Rating scale (CIRS), Activities of Daily Living (ADL), Instrumental Activities of Daily Living (IADL), Short Portable Mental Status Questionnaire (SPMSQ), Mini Nutritional Assessment (MNA), Confusion Assessment Method (CAM), body mass index (BMI), length of stay (LOS), Mini Nutritional Assessment short-form (MNA-SF), Chronic obstructive pulmonary disease (COPD), Chronic heart failure (CHF), Body mass index (BMI), Abbreviated Mental Test (AMT), 12-Item Short Form Survey (SF-12), Geriatric Depression Scale-12 (GDS, SF-12), Medical Outcomes Study (MOS), Safety Management System (VMS), Cumulative Illness Rating Scale-Geriatric (CIRS-G), Estimated Glomerular Filtration Rate (eGFR), Venous thromboembolism (VTE).

* The study does not specify the ORs of the multivariate analysis.

Van Seben et al. 2020 [38], Calsolaro et al. 2019 [31], Lehn et al. 2019 [30], Chan et al. 2019 [40], Maddox et al. 2019 [42], Cotter et al. 2012 [43], Uhlmann et al. 2017 [12], Anderson et al. 2016 [29], Bogaisky et al. 2015 [44], Fisher et al. 2016 [22], Franchi et al. 2012 [10], Ben-Chetrit et al. 2012 [47], Zapatero et al. 2012 [46], Fitriana et al. 2021 [23], Robinson et al. 2012 [3], Shu et al. 2012 [36], Higi et al. 2021 [48], Mueller et al. 2021 [27], Oud et al. 2021 [21], Wang-Hansen et al. 2022 [35], Goldin et al. 2018 [34], Kim et al. 2016 [49], Fisher et al. 2012 [41], Froom et al. 2020 [33], Scott et al. 2014 [45], Vallini et al. 2021 [28], Silber et al. 2019 [32], Finlayson et al. 2018 [11], Lin et al. 2019 [39].
